# The role of corporate social responsibility and government incentives in installing industrial wastewater treatment plants: SEM-ANN deep learning approach

**DOI:** 10.1038/s41598-023-37239-1

**Published:** 2023-10-02

**Authors:** Sohaib Mustafa, Ying Long, Sehrish Rana

**Affiliations:** 1 College of Economics and Management, Beijing University of Technology, Beijing, 100124 People’s Republic of China; 2 Office of Academic Affairs, Beijing University of Technology, Beijing, 100124 People’s Republic of China; 3 Government Islamia Graduate College for Women, Faisalabad, Pakistan

**Keywords:** Human behaviour, Disease prevention, Public health, Psychology and behaviour, Sustainability

## Abstract

Contaminated industrial wastewater is one of the severe causes of health diseases. The wastewater treatment trend in developing countries is less, and governments are not strictly pursuing the wastewater management protocols adopted by firms. To study the intention to install wastewater treatment plants at the firm level and provide policy suggestions to the developing countries’ governments, we have collected a cross-sectional dataset from manufacturing firms. We have presented an integrated model based on the theory of planned behaviour and tested our proposed model by implying SEM-ANN deep learning techniques. Results revealed that Environmental knowledge, installation cost, firm reputation, and corporate social responsibility positively influence firm management’s intention to install wastewater treatment plants, but awareness of the risk associated with contaminated wastewater has a negative influence. On the other hand, Government incentives do not influence the process. In addition, our study has found that firm size moderates the relationship between explained and exploratory variables. Our study provided valuable insight into the available literature and provided some policy suggestions to developing countries' governments to cope with water scarcity and health issues caused by contaminated industrial wastewater.

## Introduction

A good opportunity to go back and reassess fundamental notions like the need for wastewater treatment is now when so many towns are trying to cut costs and redistribute financing for public programmes. Untreated wastewater is the primary source of environmental degradation, human sickness, and death all across the globe^1^. Without managing sewage gases and smells, every town has to clean its wastewater because of the major health issues it may create^2,3^.

The use of unsafe water poses a significant risk to human health. According to the UNESCO 2021 World Water Development Report^4^, approximately 829,000 people die each year from diarrhoea that is caused by water contamination, sanitation, and hygiene practices. Among these fatalities are nearly 300,000 kids under the age of 5, which accounts for 5.3% of all deaths in this age group^5^. According to the findings of studies conducted in Palestine, residents who drink directly from municipal water sources have a higher risk of contracting illnesses such as diarrhoea compared to those who consume water that has been desalinated or filtered at home^6^. According to the findings of research that compared purified water, tap water, and bottled water, the most significant contributor to gastrointestinal sickness was tap water^7^. Helminthiasis, trachoma, cholera, and schistosomiasis, are only some of the illnesses that are more prevalent in areas where there is insufficient access to clean water and sanitation services. There is a direct link between polluted water and the spread of cholera, as shown by research conducted in underdeveloped nations; nevertheless, cholera cases may be reduced by treating and storing water in the home^8^. In addition to diseases, ingesting contaminated water and living in an unhygienic environment may cause gastrointestinal sickness, preventing proper nutritional absorption and leading to malnutrition. Children are more susceptible to the severity of these consequences.

According to the available research, some of the occupational health concerns that might be associated with the management methods of wastewater and excreta include epilepsy, skin infections, parasite infections, and bacterial infections^9^. When individuals of a community consume fish, vegetables, or fruits that have been tainted by contamination, they put themselves at risk of experiencing unfavourable health impacts^9^.

Researchers also claim that due to the chloride risk, the findings suggest that the use of treated sewerage wastewater in irrigation should be restricted to a low to moderate degree. The value of the residual sodium carbonate is more than 1.25 throughout each season, which indicates that the samples taken during the summer and fall are questionable for use in irrigation, while the samples taken during the spring and winter are inappropriate for use in irrigation^10^.

Although there may be variations in severity depending on factors such as location, gender, age, and other factors, the effects of water pollution on human health are undeniably considerable. Diarrhea is the most prevalent illness that may be attributed to water pollution. This illness is mostly caused by enteroviruses found in aquatic environments^5^.

Because of the continued growth of industrialisation and urbanisation, there is an immediate need for significant work to be done to protect water bodies from pollution and improve water quality. This is necessary in order to preserve the environment and maintain public health. In order to accomplish this objective, not only will it be necessary to create technologies that are both sustainable and effective in order to provide all communities with access to drinking water as well as sanitation and hygiene services, but it will also be necessary to implement practises that will ensure the responsible use and management of water resources.

Recent studies are mainly focusing on the technologies of water treatment plants^11–16^, the impact of clean drinking water on children under the age of five^17^, and government priorities of rural electrification over clean drinking water^18^, diseases and dangers to people's health caused by polluted water^5^, potential hazards to the environment and human health involved with the reuse of wastewater for irrigation^10^, Health risks associated with wastewater^9^. In addition, a large number of studies have led researchers to conclude that company size is an important mediator in various settings at the firm level^19,20^. Still, the role of firm size as a moderator in intentions to install wastewater treatment plants (WWTPs) and treatment of contaminated industrial wastewater is unknown. In addition to this role of awareness of risks associated with contaminated industrial wastewater, government incentives, corporate social responsibilities, and firm reputation in developing countries are also ignored in previous studies. To our knowledge, none of the studies has studied the installation intentions of management at the firm level and its associated factors or the moderation effect of firm size to understand the industry’s role in coping with the disease caused by contaminated industrial wastewater. To fill this research gap, we have proposed the following research questions.

- **RQ1**: Do environmental knowledge, government incentives, corporate social responsibility, installation cost, contaminated water risk awareness, and firm reputation play any role in installing wastewater treatment plants in the industry?
- **RQ2**: Does firm size play any moderating effect in installing wastewater treatment plants at the firm level?
- **RQ3**: What are the most critical factors behind the firm's management intentions to install a wastewater treatment plant?

To answer these research questions, we have presented an integrated model based on the theory of planned behaviour. We have collected a cross-sectional dataset from the industry managers and owners in the division Faislabad (Industrial hub of Pakistan), Pakistan. to assess their intention to install WWTPs and remove hazardous material from industrial wastewater. We have utilised the partial least square structural equation model (PLS-SEM) modelling technique to answer research questions 1 & 2 and applied the artificial neural network (ANN) deep learning approach in the second step to rank the influential factors based on their normalised importance. Results revealed that except for Government incentives, all the understudy variables explained in the research question have a statistically significant influence on the management intentions to install water treatment plants. We have also found that firm size moderates the understudy relationships. ANN sensitivity results revealed that corporate social responsibility is the most significant influential factor that motivates firm management to install WWTPs.

## Conceptual framework

**Figure 1:**
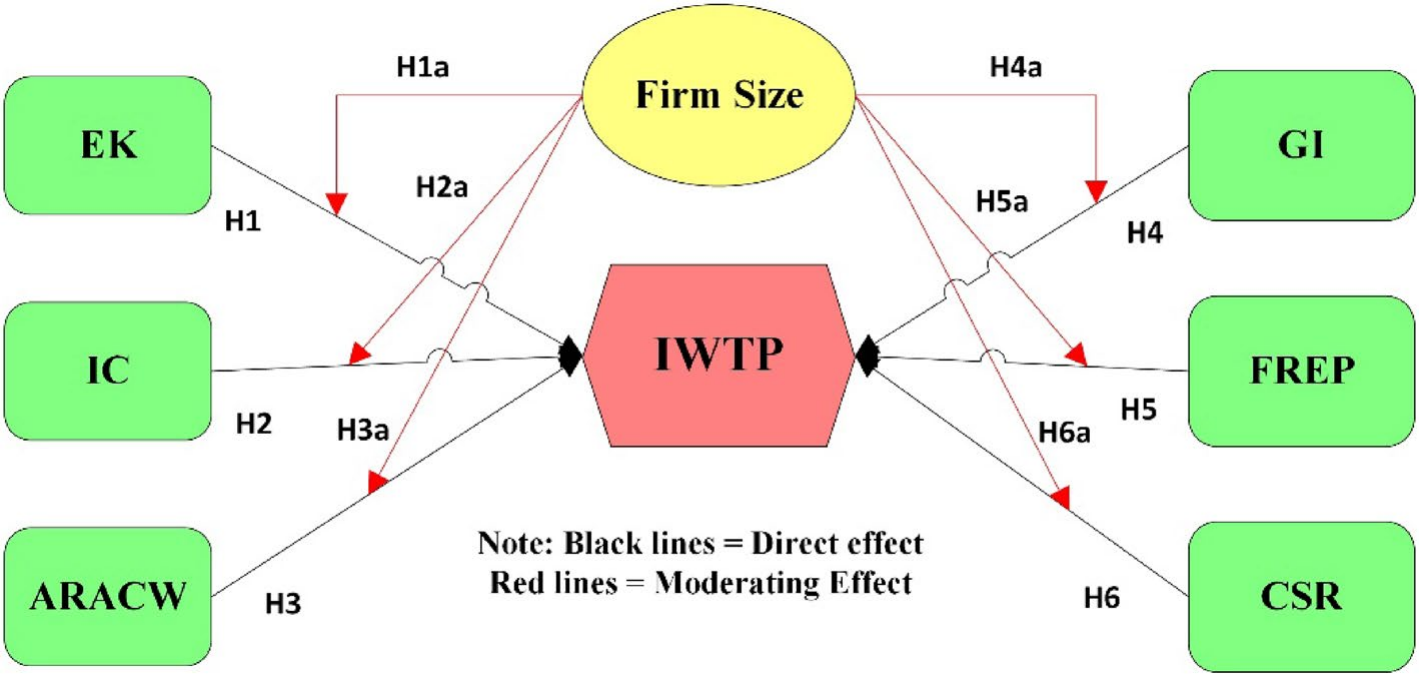
Conceptual framework. *IWTP* intention to install water treatment plant, *EK* environmental knowledge, *IC* installation cost, *FREP* firm reputation, *ARACW* awareness of risk associated with contaminated water, *GI* Government incentives, *CSR* corporate social responsibility.

The theory of planned behaviour (TPB), a cognitive theory, developed by Ajzen^21^, states that the choice to participate in certain conduct might be dependent on the individual's desire to engage in that particular behaviour. “Intentions are assumed to capture the motivational factors that influence behaviour; they are indications of how hard people are willing to try, of how much of an effort they are planning to exert in order to perform the behaviour. As a general rule, the stronger the intention to engage in a behaviour, the more likely its performance should be”^21^. Based on this cognitive theory, we have proposed an integrated model (Fig. 1) presented below. We postulate that environmental knowledge, installation cost, firm reputation, awareness of the risk associated with contaminated water, government incentives, and corporate social responsibility will shape a firm's management behaviour in installing WWTPs.

### Pakistan and water scarcity

There will be a "serious water deficit" in Pakistan by the year 2025, according to a study that was compiled by the "Pakistan Academy of Science and the Council for Research in Water Resources" (PCRWR)^22^. It is estimated that just around twenty percent of the total population of Pakistan has access to potable water. Because there are not enough sources of clean and healthy water supply, the remaining 80 percent of the population is being forced to utilise water that is not fit for human consumption^23^. The most common form of pollution is sewage, sometimes known as faeces, which is frequently released into the sources of drinking water systems^23^. According to the International Monetary Fund (IMF), Pakistan is the third most water-scarce country in the world (on the water scarcity index, which evaluates nations based on the proportion of their total annual withdrawals compared to their national annual water resource)^24^. Hence it is essential for countries like Pakistan to clean the wastewater for reuse and to avoid as much health and economic loss as it can. Unfortunately, the industrial sector in Pakistan is not paying much attention to the reuse of water or is much concerned about the diseases caused by industrial wastewater.

Because Pakistan is rapidly becoming a nation with a water shortage, it is imperative that we think logically and devises some non-traditional methods for recycling water so that it may be used for various reasons, such as washing clothes, cleaning homes, and so on. Processes that are chemical, physical, and biological in nature are used in sewage treatment plants in order to purify the water so that it may be used in a manner that is not only safe but also beneficial to human health. STP Plants are also responsible for the treatment of chemicals and dangerous pollutants that are found in the wastewater that is produced by industries and other commercial enterprises.

## Hypothesis development

### Environmental knowledge and intention to install water treatment plant

Environmental knowledge is the awareness, comprehension, and application of information pertaining to environmental problems and potential remedies^25^. To have environmental knowledge is to seek out environmental information and awareness in order to address environmental challenges and develop solutions for such concerns^26^. In addition, improving awareness and understanding of environmental issues and providing people with the opportunity to contribute information or views about environmental issues facilitates the making of responsible decisions^27^. Environmental knowledge is found to be a significant factor behind the adoption and use of products that are eco-friendly and help to improve the ecosystem^25^. Researchers have also found that students with better environmental knowledge are more inclined toward waste management^28^. Researchers also claimed that knowledge of contaminated water positively shapes public awareness, which leads to the intention to use clean water^29,30^. It helps to understand the environmental risks and remedies that can minimise environmental damage. We suppose that environmental knowledge will positively influence the management of firms to treat their wastewater to minimise the expected environmental damage and associated health risks.

- **H1**: Firm management’s environmental knowledge positively influences the intention to treat wastewater and install water treatment plants.

### Installation cost and intention to install water treatment plant

Installation cost refers to the investment/expense a firm bears to install a plant or machinery in the industry for operations. And the concept of cost value refers to the investment/expense a firm made to install a piece of machinery compared to the expected or actual benefits it will render to the firm after its installation. Researchers have found both positive and no association of cost with a certain outcome in the past. For example, it positively influences the intention^25^ or no effect^31^. Some studies have also considered the cost factor as a barrier to installing or adopting new technology^32^. In this study, we anticipate that installation cost might influence the installation intention of firm management. Hence we have hypothesised the following.

- **H2**: The installation cost of WWTPs positively influences the firm’s management intention to install WWTPs.

### Awareness of risk associated with contaminated water and intention to install water treatment plant

People's acceptance or rejection of technological innovation is dependent on their degree of awareness, which may be described as their knowledge or acknowledgement of the advantages and downsides of the invention/technology^25^. Prior to this study, few academics have investigated why people choose to install industrial sewage treatment plants. According to several studies, many individuals are unaware of the benefits of employing water filtering systems in their homes or workplace to prevent illnesses caused by industrial wastewater^3^. Researchers have revealed that low awareness of risks associated with different types of cancer plays an influential role in fighting against the disease^33,34^. A study of residential community college students found that those with greater knowledge of mental health disorders were more likely than those with lower knowledge to suggest supportive resources (such as a trip to the counselling centre) to a friend who was showing signs of a mental health disorder^35^. We assume that the more knowledgeable a firm’s management is about the risk associated with contaminated water and WWTPs, the more likely they are to adopt them. Consequently, we'll make the following assumptions.

- **H3**: Awareness of the Risk associated with contaminated water will influence the firm’s management to install a WWTP.

### Government incentives and intention to install water treatment plant

Government incentives refer to the subsidies and other tax relaxation of benefits provided by any government to its residents or firms operating within the jurisdiction of a specific government. Researchers have found that incentives provided by governments have an influential role in the consumers’ or stakeholders’ decision-making in different fields. For example, a study in China revealed that Government incentives have a positive association with enterprises' innovation performance when there is financial slack; however, the research finds an inverse link between government incentives and innovation performance when there is human slack^36^. Another study found that under the incentive strategy of total investment in research and development that leads to better environmental improvement, the greening level has been increased to its highest possible level. Members of the supply chain may be willing to make concessions regarding their environmentally friendly objectives to increase their profitability^37^. Researchers in China have also discovered that technical development is still a factor that is holding back the new energy vehicle sector and that technological advancement's influence on the spread of new energy vehicles is bigger than the effect of economic subsidy policy^38^. On the contrary, the findings indicate that a discount on the initial purchase price of an electric vehicle is the one-time financial incentive that consumers most desire. This is particularly the case in Australia, where EVs are anticipated to be rather pricey^39^. In conclusion, as there is no sufficient literature available on the government incentives’ role in the firm decisions to instal WWTPs, we assume that it will affect the management to install contaminated WWTPs.

- **H4**: Government incentives will influence the firm’s management's intention to install a water treatment plant.

### Firm reputation and intention to install water treatment plant

The term “firm reputation” refers to the consensus of public opinion of a certain business or organisation. It takes into account aspects such as the results of search engines, the coverage provided by the news, and the activities of the firm that are made public. It is also considered an intangible asset of any business. A company’s reputation may be described as the consumers' opinions of how effectively a company cares for its customers and how genuinely concerned it is about the well-being of its clients^40^. The firm reputation is also responsible for public investment and share purchases, so it is a vital factor that a firm’s management is concerned about sustaining in the market. Every firm’s management is conscious of its reputation and does all the actions that can enhance its reputation. According to the findings of a study in Indonesia, a company’s environmental performance has a favourable and noticeable impact on the reputation of the business^41^. We assume that a firm's reputation is important for firm survival, so firm management will treat its contaminated industrial wastewater to enhance its reputation. Therefore we have hypothesis the following.

- **H5**: Firm reputation will positively influence the firm’s management in the installation of WWTPs.

### Corporate social responsibility and intention to install water treatment plant

Corporate social responsibility (CSR) activities may be a significant part of public relations for corporations. The actions and policies implemented by organisations with the intention of having a beneficial effect on the world are referred to as corporate social responsibility, or CSR for short. The core tenet of corporate social responsibility is the belief that businesses should work toward a variety of altruistic goals in addition to maximising their profits. Common corporate social responsibility goals include reducing a company's negative impact on the surrounding environment, encouraging workers to participate in community service, and making charitable contributions. Researchers have found that corporate social responsibility has a major beneficial impact on a firm's reputation and performance, but it has a negative effect on the firm's risk. According to the findings, they further affirm that a firm's reputation does, in fact, play the role of a mediator in the connection between corporate social responsibility-firm performance and corporate social responsibility-firm risk^42^. Researchers have also discovered that company reputation is favourably associated with business performance while adversely related to firm risk, and they establish a significant positive correlation between corporate social responsibility and firm reputation^43^. With this literature, we assume that CSR will positively influence management to treat their contaminated industrial wastewater and improve their reputation in the market.

- **H6**: Corporate social responsibility positively influences the firm’s management to treat their wastewater and install the WWTP.

### Firm size as moderator

Firm size plays a decisive role in many decisions performed by management. Financial condition, employee headcount, and related factors are key components in determining the size of a firm. It has been revealed in many studies that firm size is a significant mediator in a different context at the firm level, such as^19,20^. The installation of a WWTP to treat industrial wastewater is a firm investment that will enhance its reputation and fulfil its social responsibility. It is also expected that firm size will play a decisive role in management awareness, the seriousness of risks associated with draining industrially contaminated wastewater, and Government incentives announced to help the industry cope with the disease caused by industrial wastewater. The moderating role of firm size in treating industrial-contaminated wastewater and installing WWTPs is unknown. Hence, we have hypothesised the following.

The firm size will moderate the relationship between Environmental knowledge **(H1a)**, Installation Cost **(H2a)**, Awareness of Risk associated with contaminated water **(H3a)**, Government incentives **(H4a)**, Firm Reputation **(H5a)**, and Corporate social responsibility **(H6a)** with the intention to install water treatment plant.

## Research methodology

### Questionnaire design

In order to study the research topics, surveys were administered to the managers and owners of both small and large firms in the division of Faisalabad, which is located in central Punjab in Pakistan, a leading industrial division. In order to verify the content validity of the measurements, every measurement item from every current instrument was modified to fit the context of technology adoption. The questionnaire contained a total of 22 items that were connected to the constructs that were being investigated. On a Likert scale with five points, where 1 represents “strongly disagree”, and 5 represents “strongly agree”, each factor was evaluated using this scale.

In order to assure the reliability and validity of the instrument, a two-stage preliminary test of the questionnaire was carried out. In the beginning, there were three professors who looked over the questionnaire to ensure that there was no ambiguity in the phrasing or format, that it was easy to comprehend, that it adhered to logic, and that it was relevant to the context. Based on the suggestions offered by the professors, certain phrasing and the order of the items were modified somewhat. After then, a preliminary test was carried out. Ten “Faisalabad industrial estate development & management company” members filled out the amended questionnaire and returned it to the company. It was requested that suggestions and criticisms be provided on the instrument’s structure and elements. The responses from these professionals were considered while revising the questionnaire. The constructions of the survey, as well as the items, are listed in the Supplementary Material.

The utilization of quantitative analysis is a common practice in scholarly articles as it enables the measurement of human behavioral intentions through the provision of dependable and unbiased data. Quantitative research is grounded on empirical data that can be obtained through surveys, interviews, or experiments and employs statistical techniques to scrutinize the data^44^. The aforementioned research methodology is valuable in precisely assessing human behavior as it enables researchers to observe and quantify the actions of a substantial sample size and to extrapolate the results to a broader population^44^. Moreover, the utilization of quantitative analysis enables the examination of hypotheses and the detection of associations among variables^42^. The aforementioned approach facilitates the discernment of fundamental factors that drive behavior, a task that may prove challenging to accomplish through qualitative methodologies^45^.

### Data collection

**Figure 2:**
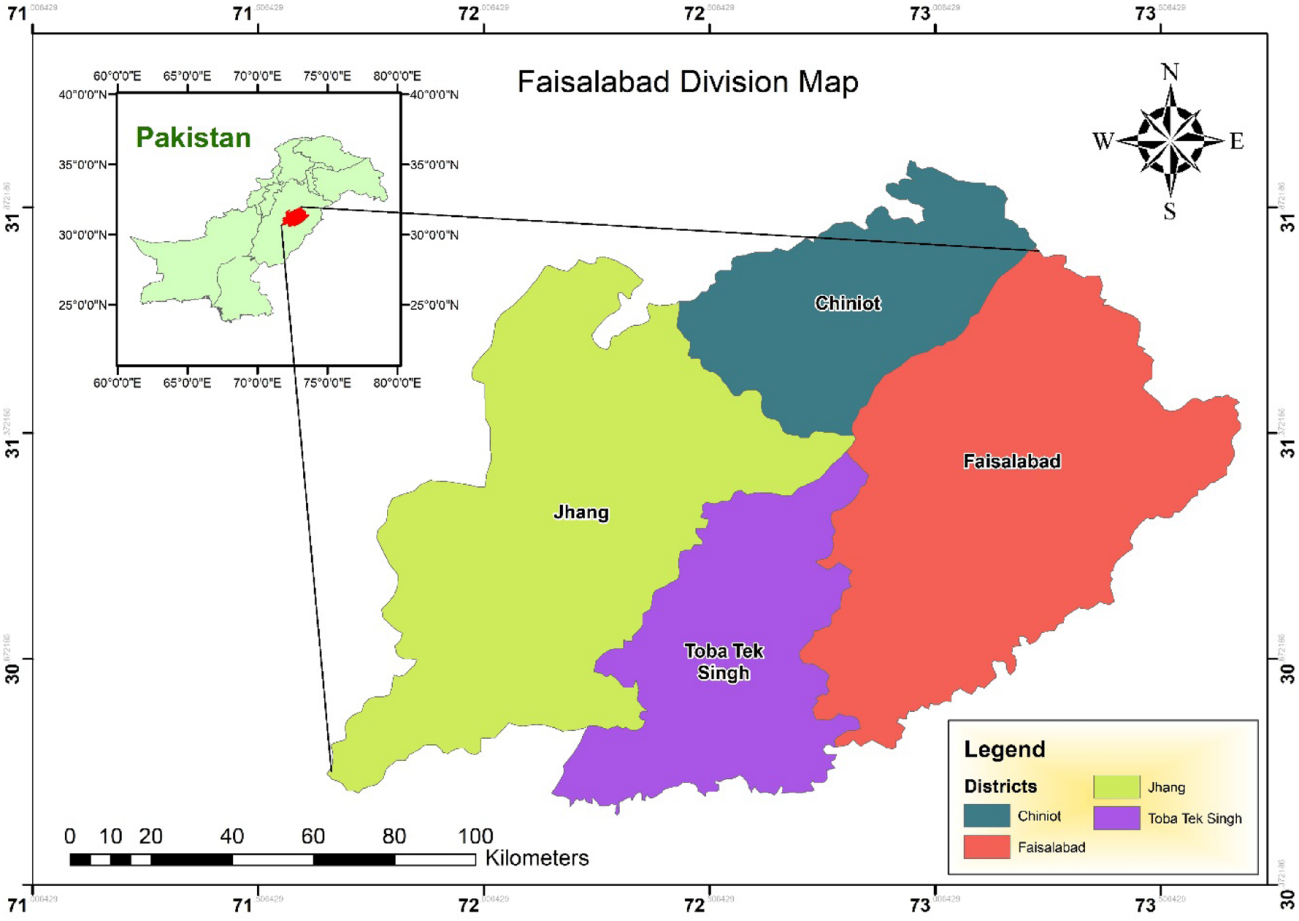
Study area: Authors have downloaded a free version of the ArcGIS shapefile of Pakistan available at https://data.humdata.org/ and used ArcGIS version 10.8.1 to incorporate legend. Te legend shows the name of Pakistan cities where the survey was conducted to collect the dataset for analysis.

Following the completion of the pilot project, empirical data were gathered via an in-person survey. The survey team consisted of fifteen students working toward their doctorates, and three research associates visited the companies in person and gathered the data. The data utilized by the researchers was obtained from the censuses of Pakistan’s manufacturing sector, which the Pakistan Bureau of Statistics conducted during the period of 2015–2016. The investigators employed a simple random sampling technique as it guaranteed that each element in the research had an identical probability of being chosen. The results have the potential to be generalized to a broader demographic. The companies that have their headquarters in the Faisalabad Judicial Division are contacted for data collection (Fig. 2). Gathering datasets from the responsible management or owners of the companies is the operation's goal. In addition, we made certain that all replies would be kept strictly secret. The survey was conducted for two months, beginning in April 2022. A total of 650 questionnaires were distributed. A total of 482 replies were received to the survey, with a response rate of 74.15%. We gave our complete attention to each questionnaire that was handed back in. There are 265 small firms and 217 large corporations. We have used the definition supplied by suggestion 2003/361 issued by the EU [https://ec.europa.eu/transparency/documents-register/detail?ref=SWD(2021)279&lang=en] to segregate small and large firms and OECD.org^46^. The demographic information of the respondents that were considered legitimate is shown in Table 1.

**Table 1:** Demographic characteristics.

Characteristics	Distribution	Frequency
Respondent’s gender	Female	140
	Male	342
Respondent’s designation	Owner	205
	Manager	275
	First line staff	02
Respondent’s education	PhD	05
	Masters	359
	Bachelor	118
	Undergraduate	–
Industry	Manufacturing	482
	Services	–
Firm age	Less than 5 years	50
	5–10 year	106
	10–15 year	176
	More than 15 years	150
Employees	Less than 50	265
	More than 50	217

We investigated the non-response bias by analysing the replies obtained in the early (i.e. the first 15 days) and late phases of the survey. This allowed us to check for the self-selected character of the response as well as the external validity of the study (i.e. the last 15 days). According to the findings, the degrees of significance for intention to install a water treatment plant, Environmental Knowledge, Installation Cost, Firm Reputation, Awareness of Risk associated with contaminated water, Government incentives, and Corporate social responsibility are as follows: p = 0.232, 0.254, 0.458, 0.623, 0.896, and 0.756 correspondingly. Because the level of significance of all constructs is more than 0.05, it can be deduced that there is no difference between the replies obtained in the early and late phases of the research and that non-response bias is not a factor in this investigation.

The “Mann–Whitney and Kruskal–Wallis” tests were carried out in order to investigate whether or not there was a significant difference in the replies received from the two categories of firms. A study of the demographics of two different kinds of firms based on their size revealed no differences. As a result, the method bias was found to be insignificant, and the collected data may now be analysed. In addition, Harmon's one-factor test was carried out in order to establish whether or not a common method bias was present. The whole set of seven latent variables was subjected to exploratory factor analysis. According to the findings, a single component can only explain a limited amount of variation (less than thirty percent). Therefore, the effect of the common method bias on our sample was minimal.

### Data analysis and results

PLS-Smart 3 was used for the purpose of doing the data analysis, while a structural equation model was utilised in order to evaluate the hypotheses. Two different methods were used in order to examine the data. First, an analysis of the validity and reliability of the measurement was carried out. In the second step of the process, an analysis of the structural connections between two latent variables was carried out.

#### Measurement model

The reliability of the measurement model's constructs, as well as their discriminant and convergent validity, were assessed and found to be satisfactory for validation purposes. Researchers^47,48^ proposed the use of composite reliability (CR) as a method for evaluating construct reliability. According to these authors, the values of CR should be more than 0.7. According to Table 2, all of the constructions exhibited a high level of reliability. In addition to that, Cronbach’s alpha (α) was determined. According to Table 2, the Cronbach's alpha for each construct was more than 0.841, which is greater than the required threshold of 0.7^49,50^. Therefore, the constructs used in this investigation have a satisfactory level of reliability.

**Table 2:** Reliability and validity analysis.

Constructs	Items	Loadings	T statistics	VIF	CR	α	AVE
Awareness of risk associated with contaminated water	ARACW1	0.855***	48.129	2.207	0.918	0.881	0.736
	ARACW2	0.877***	50.309	2.432			
	ARACW3	0.864***	54.864	2.159			
	ARACW4	0.835***	33.735	2.248			
Corporate social responsibility	CSR1	0.859***	52.728	1.955	0.918	0.865	0.788
	CSR2	0.893***	77.656	2.425			
	CSR3	0.911***	106.149	2.608			
Environmental knowledge	EK1	0.868***	47.29	2.092	0.908	0.847	0.766
	EK2	0.887***	47.162	2.257			
	EK3	0.870***	50.203	1.889			
Firm reputation	FREP1	0.700***	19.258	1.184	0.841	0.714	0.640
	FREP2	0.826***	35.305	1.764			
	FREP3	0.865***	48.64	1.831			
Government incentives	GI1	0.872***	57.802	1.82	0.891	0.818	0.731
	GI2	0.847***	38.604	1.953			
	GI3	0.846***	40.663	1.723			
Installation cost	IC1	0.886***	64.589	2.083	0.904	0.840	0.757
	IC2	0.865***	37.887	2.112			
	IC3	0.860***	42.637	1.83			
Intention to install a water treatment plant	IWTP1	0.819***	37.651	1.617	0.871	0.779	0.693
	IWTP2	0.848***	47.16	1.653			
	IWTP3	0.830***	43.461	1.566			

α > 0.7; CR > 0.7; AVE > 0.5; VIF < 5; ***Significant at p < 0.001; Cronbach’s alpha: α.

**Figure 3:**
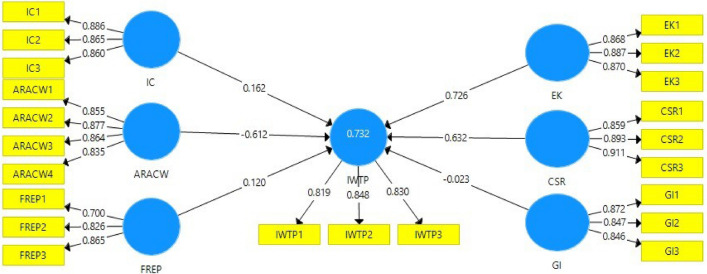
Measurement model.

Two criteria may be used to determine whether or not the scales have convergent validity^47,51–54^. First, the loading of every construct item that has to be considered in the analysis needs to be more than 0.7 with a p-value less them 0.05 (Fig. 3). Second, in order to reduce the possibility of measurement error, the average variance extracted (AVE) value should be greater than 0.5. The results shown in Table 2 demonstrate that each factor loading is significant and is above the acceptable threshold of 0.7, while the AVE for the constructs ranges from 0.640 to a high of 0.788. As a result, our model exhibits convergent validity that is up to par.

The concept of “discriminant validity” refers to the extent to which the measures of two independent concepts were discovered to be empirically distinct from one another. It was discovered that the square root of the AVE for each construct was higher than the correlations between the constructions and all of the other constructs. If the square root of the AVE for each construct was more than the correlation between constructs, then this implies that the constructs are discriminantly relevant with respect to one another, and this is the case if the correlation between constructs was greater than the AVE^47,55^. Consequently, the findings demonstrated that the discriminant validity met the requirements (Table 3).

**Table 3:** Discriminant validity (Fornell-Larcker criteria).

	STD	Mean	ARACW	CSR	EK	FREP	GI	IC	IWTP
ARACW	1.030	3.777	**0.858**						
CSR	0.929	3.990	0.380	**0.888**					
EK	1.052	3.751	0.989	0.384	**0.875**				
FREP	0.949	3.885	0.475	0.631	0.485	**0.800**			
GI	0.874	4.009	0.559	0.503	0.552	0.553	**0.855**		
IC	0.816	4.130	0.414	0.442	0.413	0.472	0.368	**0.870**	
IWTP	0.845	3.949	0.457	0.813	0.476	0.643	0.479	0.536	**0.832**

Bold diagonal values are the square root of AVE.

*IWTP* intention to install water treatment plant, *EK* environmental knowledge, *IC* installation cost, FREP firm reputation, *ARACW* awareness of risk associated with contaminated water, *GI* government incentives, *CSR* corporate social responsibility, *STD* standard deviation.

#### Structural model (PLS-SEM)

The SEM technique was selected because it allows the assessment of data with different sample sizes, enables the examination of subgroups, and facilitates the testing of complicated structural models with various components. These are the reasons why the SEM method was chosen. In this particular research, there were a total of six independent variables that were to be evaluated using twenty-two different questions. In order to identify the dependent variables, we formulated a total of six hypotheses.

The SEM was used to evaluate the IWTP, which is the method that is best suited for exploratory work because of its theoretical backing for ideas and its small sample^3,56,57^. The likelihood of their being multi-collinearity across several independent manifestations is quite high due to the fact that there are many data sets pertaining to the constructs. The SEM procedure is useful because it presupposes that specific effects are getting better one at a time with the remainder of the constructs in the data analysis frame. This presumption is what makes the SEM procedure so helpful. The measurement phase of the model is where the ensuing outcome signals are looked at and analysed^57,58^. SEM, as opposed to CB-SEM, employs proxies for latent variables in order to account for error terms. This is due to the fact that the model does not directly deal with hidden variables. The researchers decided to employ the structural approach rather than the regression technique for this experimental study's sample since the sample size was small. The R^2^ value for the dependent construct was used in the analysis to determine how well the models explained the data, and Q^2^ for predictive relevance (Table 4). To run the direct path model, we have used 5000 resampling in bootstrapping as suggested by earlier researchers^25,26,51,56^.

**Table 4:** Direct paths.

Statistical paths	β	Std. dev	T-value
ARACW→IWTP	− 0.602***	0.220	2.741
CSR→IWTP	0.631***	0.044	14.24
EK→IWTP	0.707***	0.224	3.162
FREP→IWTP	0.122***	0.042	2.887
GI→ IWTP	− 0.025^NS^	0.044	0.553
IC→IWTP	0.17***	0.036	4.789
R^2^	0.733		
Adjusted R^2^	0.730		
Q^2^	0.498		
Normed fit index (NFI)	0.953		

***Significant at p < 0.001, **Significant at p < 0.05.

*NS* not supported, *IWTP* intention to install water treatment plant, *EK* environmental knowledge, *IC* installation cost, *FREP* firm reputation, *ARACW* awareness of risk associated with contaminated water, *GI* government incentives, *CSR* corporate social responsibility, *STD* standard deviation.

**Figure 4:**
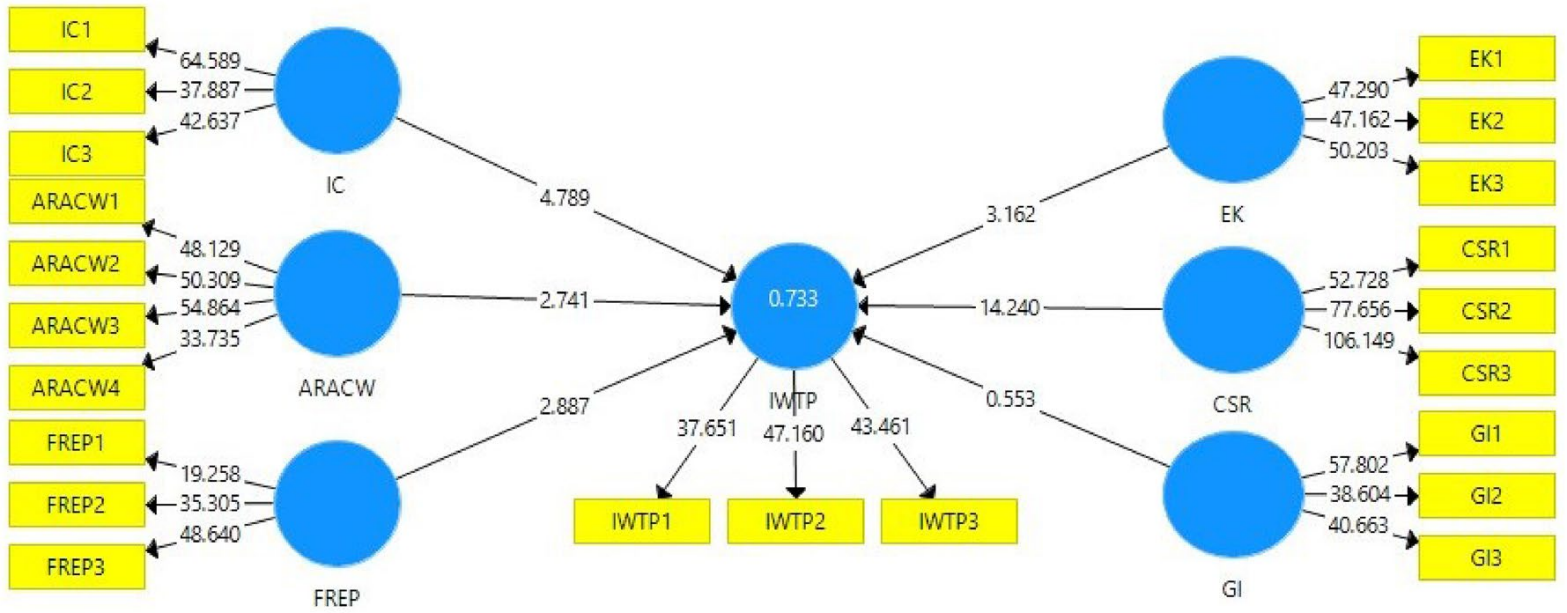
Path model.

The findings of our hypothesised model are shown in Fig. 4, including the R^2^ value and the *t* values. The fact that the model was able to explain 73% of the variation in respondents' intentions to install water treatment plants is evidence that the model had adequate explanatory power^59,60^. The standardised path coefficients of Awareness of Risk associated with contaminated water (β = – 0.602, p = 0.006), Corporate social responsibility (β = 0.631, p < 0.001), Environmental Knowledge (β = 0.707, p = 0.002), Firm Reputation (β = 0.122, p = 0.004), and Installation Cost (β = 0.17, p < 0.001) with an intention to install water treatment plant were all significant. Therefore, H1 to H3 and H5 and H6 are accepted. There was no evidence found to support hypothesis H4.

#### Moderation analysis

In addition to the direct path model assessment, we have run a separate model with the same bootstrapping sample to test the moderation effect of firm size. According to the findings in Table 5, firm size acts as a moderator in a positive way for the relationships between ARACW, CSR, EK, and IWTP. In contrast, it acts as a moderator in a negative way for the link between IC and IWTP. The second model’s direct effect findings remain true, demonstrating the resilience of model 1’s findings. Hence, with the results presented in Table 5, H1a, H2a, H3a, and H6a are accepted, but we rejected H4a and H5a.

**Table 5:** Moderation analysis.

Statistical paths	β	Std. dev	T-value
ARACW→IWTP	0.438**	0.136	2.328
CSR→IWTP	0.138***	0.163	14.16
EK→ IWTP	0.823***	0.121	2.137
FREP→IWTP	0.086**	0.134	2.401
GI→IWTP	− 0.031^NS^	0.118	0.659
IC→ IWTP	0.323***	0.146	2.935
FS*ARACW→IWTP	0.431***	0.072	3.459
FS*CSR→IWTP	0.390***	0.084	2.979
FS*EK→IWTP	0.529**	0.104	2.891
FS*FREP→ IWTP	0.141^NS^	0.136	0.325
FS*GI→IWTP	0.231 ^NS^	0.102	0.553
FS*IC→IWTP	− 0.096**	0.105	2.479

***Significant at p < 0.001, **Significant at p < 0.05.

*NS* not supported, *FS* firm size, *IWTP* intention to install water treatment plant, *EK* environmental knowledge, *IC* installation cost, *FREP* firm reputation, *ARACW* awareness of risk associated with contaminated water, *GI* government incentives, *CSR* corporate social responsibility, *STD* standard deviation.

**Figure 5:**
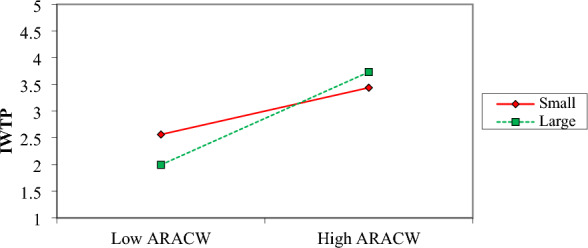
Moderation effect of firm size on ARACW and IWTP.

The moderating effect that the size of the firm has on the correlation between ARACW and IWTP is seen in Fig. 5. When compared to small enterprises, big firms have far more pronounced and positive gradients, which are shown in the plot. As a result, this demonstrates that the influence of ARACW in persuading management to set up a water treatment facility is more significant in larger companies.

**Figure 6:**
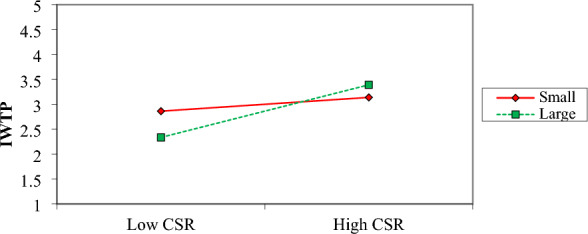
Moderation effect of firm size on CSR and IWTP.

The same is the case with CSR. The moderating effect that the size of the firm has on the correlation between CSR and IWTP is seen in Fig. 6. When compared to small enterprises, big firms have far more pronounced and positive gradients, which are shown in the plot. As a result, this demonstrates that the influence of CSR in persuading management to set up a water treatment facility is more significant in larger companies. Hence, large firms are more concerned about corporate social responsibility.

**Figure 7:**
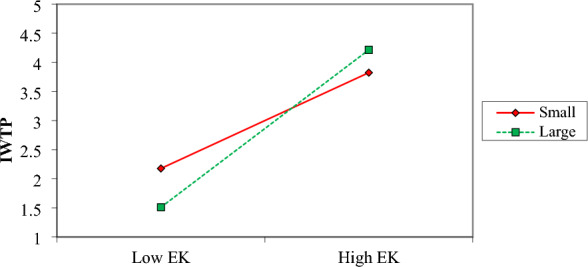
Moderation effect of Firm size on EK and IWTP.

Firm size also moderates the influence of environmental knowledge on the intentions to install water treatment plants (Fig. 7). Big firms have a stronger influence on environmental knowledge compared to small firms. Big firms are more inclined toward installing water treatment plants in response to better environmental knowledge.

**Figure 8:**
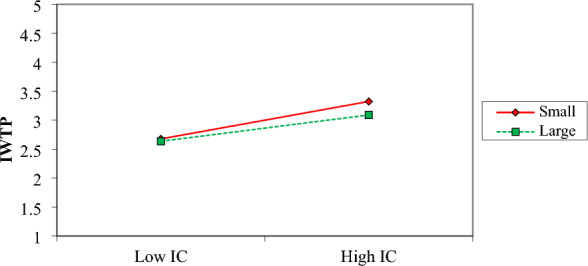
Moderation effect of Firm size on IC and IWTP.

The installation cost of the water treatment plant is a stronger influential factor for small firms compared to large ones (Fig. 8). The slope for small firms is steeper compared to large firms. It means as much as the installation cost is higher, the intention to install a water treatment plant is less in small firms.

### ANN for prediction of intention to install water treatment plant

The capabilities of the SEM tool are restricted to the identification of linear connections and the reduction of the complexity of the decision-making process^25,52^. It oversimplifies the intricacies inherent in the decision-making process that was brought about by the circumstances. Therefore, it is insufficient to simulate more complicated connections. An artificial neural network (ANN), which is a technology used in AI, may be used to represent complicated situations with linear and non-linear interactions in order to solve this issue. Unlike SEM, the ANN model takes a non-compensatory approach and does not need distribution assumptions such as linearity, normality, or homoscedasticity^51,56,61^. In addition to this, ANN models are more resilient, responsive, and accurate than traditional statistical approaches^56^. Due to the fact that the ANN approach is conducted inside a black box, it cannot be used for testing the hypothesis or establishing the causal link^25,52^. Thus, a non-linear and non-compensatory ANN technique should be used in conjunction with a linear and compensatory SEM model.

**Figure 9:**
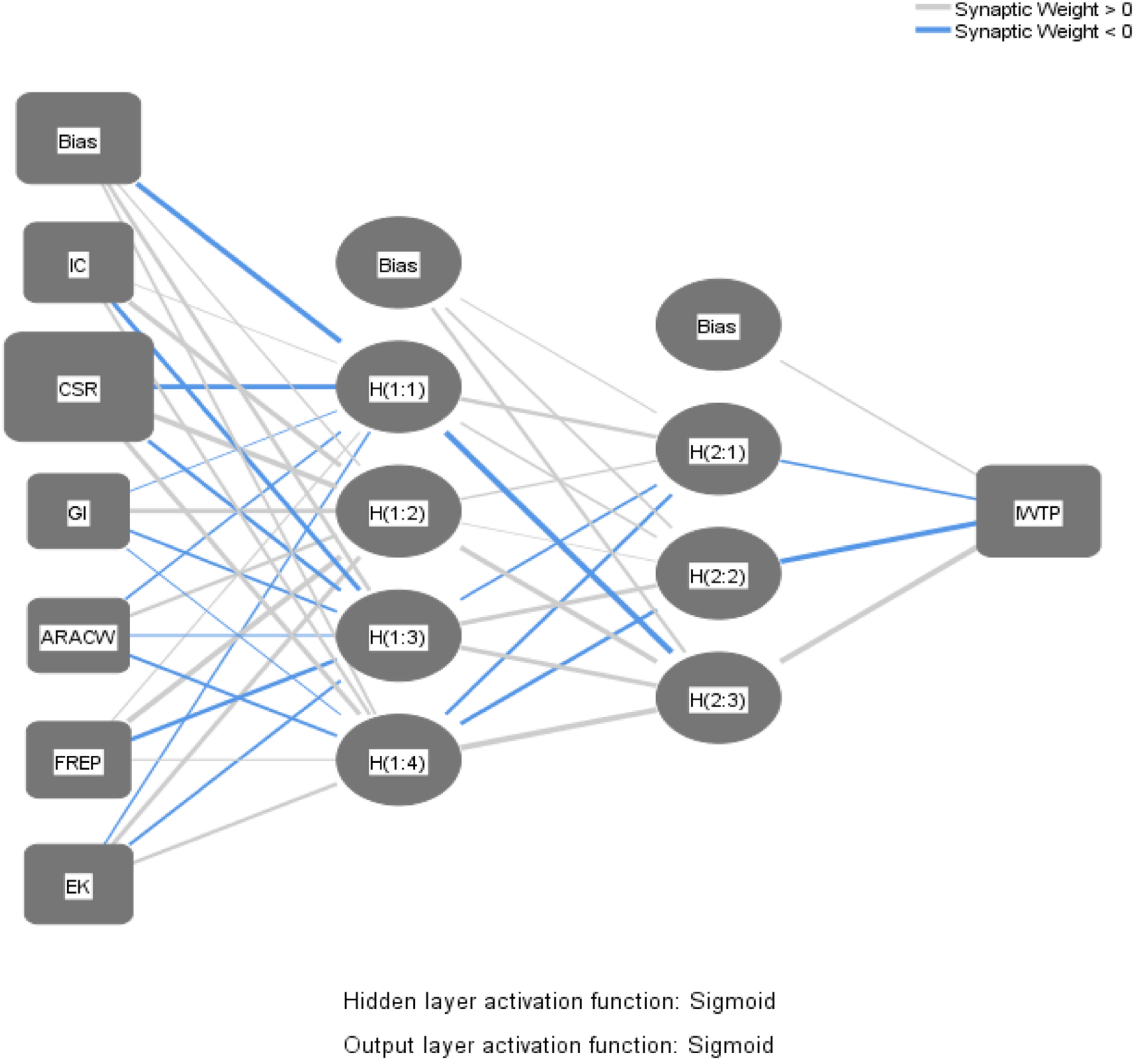
ANN model for IWTP.

One layer is the input, two are the concealed layer, and the last layer is the output layer (Fig. 9). To prevent issues with overfitting, SPSS 20.0 is used in conjunction with a tenfold validation that uses a data split of 90:10 for training and testing^25,52^. The feedforward ANN is responsible for the forward propagation of signals from the input layer all the way to the output layer of the network. Learning is done in a controlled environment to teach the network. The term “supervised learning” refers to the process of storing information in a network and repeatedly exposing that knowledge to patterns of inputs and outputs that have been established. The number of hidden neurons present in the layer containing the inputs is equal to the total number of inputs, also known as the Factors. On the other hand, the number of neurons that are present in the layers that output is equal to the number of outputs, which is the dependent variable. Due to the lack of any definite heuristic technique, the frequency of hidden neurons is chosen by the process of trial and error. This number impacts both the speed and the accuracy of the training process^56^. The non-zero synaptic weights that are coupled to the hidden layer of the artificial neural network (ANN) provide evidence that the predictor is significant^52^.

**Figure 10:**
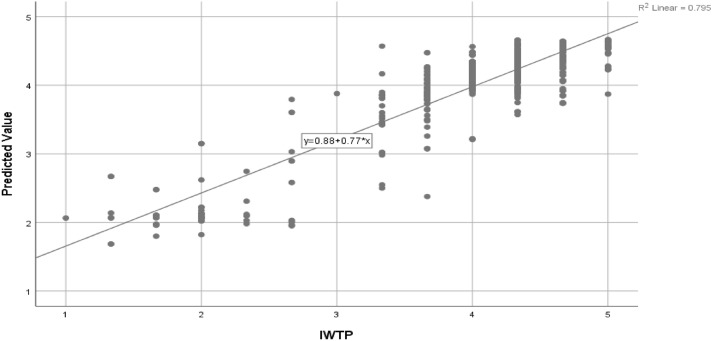
Regression standardised residuals.

The model of the neural network is presented in Fig. 9. The sigmoid function acts as an activation function for the hidden layer and the layer representing the model’s output^25^. The root means square error (RMSE) is computed for training and testing for each of the 10 neural networks in order to provide an evaluation of the accuracy of the model's prediction capabilities. Table 6 contains the calculated mean as well as the standard deviation value for the RMSE. The mean RMSE is quite low (see Table 6 for more information; it is equal to 0.073 for training and 0.072 for testing), which shows that the neural network is reliable and that correct prediction may be made^52^. Additionally, the effectiveness of the ANN models was assessed by constructing a goodness-of-fit coefficient using a predetermined method. Models of regression make use of a coefficient known as R^2^ that has a value comparable to this (Fig. 10).

**Table 6:** RMSE values for training and testing.

Training			Testing			Total sample
N	SSE	RMSE	N	SSE	RMSE	
431	2.351	0.074	51	0.312	0.078	482
431	2.261	0.072	51	0.180	0.059	482
421	2.032	0.069	61	0.304	0.071	482
426	2.245	0.073	56	0.273	0.070	482
437	2.294	0.072	45	0.256	0.075	482
429	2.135	0.071	53	0.292	0.074	482
434	2.792	0.080	48	0.255	0.073	482
425	1.870	0.066	57	0.298	0.072	482
430	2.217	0.072	52	0.363	0.084	482
435	2.612	0.077	47	0.174	0.061	482
Mean	2.281	0.073	Mean	0.271	0.072	
Std dev	0.265	0.004	Std dev	0.058	0.007	

*R*^2^ = 1-RMSE/S^2^, where S^2^ is the variance of the test data's desired output.

N number of samples. *RMSE* root mean square of errors, *EK* environmental knowledge, *IC* installation cost, *FREP* firm reputation, *ARACW* awareness of risk associated with contaminated water, *GI* government incentives, *CSR* corporate social responsibility, *IWTP* intention to install water treatment plant served as the output neuron.

#### Sensitivity analysis

The sensitivity analysis presented in Table 7 examines the significance of the values of exploratory variables, also known as factors, and the values predicted by the ANN model change depending on the factors. The normalised importance calculates the ratio of the significance of each element to the important value considered the most significant. According to the findings shown in Table 7, it has been determined that Corporate social responsibility (CSR) is the most important factor behind the intention to install a water treatment plant. In comparison to CSR rest of the factors are not much important for firm management installing WWTPs.

**Table 7:** ANN model for IWTP (sensitivity analysis).

Neural network	IC	CSR	GI	ARACW	FREP	EK
NN-1	0.128	0.645	0.027	0.023	0.063	0.114
NN-2	0.124	0.615	0.027	0.052	0.114	0.069
NN-3	0.108	0.474	0.035	0.077	0.082	0.223
NN-4	0.126	0.623	0.035	0.022	0.069	0.125
NN-5	0.085	0.567	0.023	0.064	0.079	0.182
NN-6	0.098	0.611	0.020	0.034	0.100	0.137
NN-7	0.172	0.545	0.056	0.012	0.089	0.126
NN-8	0.157	0.529	0.020	0.052	0.129	0.114
NN-9	0.128	0.630	0.044	0.029	0.050	0.119
NN-10	0.152	0.535	0.048	0.052	0.108	0.105
Average importance	0.128	0.577	0.033	0.042	0.088	0.131
Normalised importance	22.1%	100%	05.7%	07.2%	15.3%	22.7%

*EK* environmental knowledge, *IC* installation cost, *FREP* firm reputation, *ARACW* awareness of risk associated with contaminated water, *GI* government incentives, *CSR* corporate social responsibility.

Environmental knowledge and installation costs have almost identical importance. Whereas Awareness of Risk associated with contaminated water (ARACW) and Government incentives (GI) are the least important factors for firm management.

### Research involved in Human and animal rights

We confirm that all methods were carried out in accordance with relevant guidelines and regulations. Humans who participated in this study are aware of the purpose of the study, and their confidential information has not to be shared with anyone.

### Informed consent

All study participants provided their written informed consent. Study data is used after the consent of participants. The questionnaire used in this study started with the declaration and purpose of the study.

### Ethical approval

The experimental protocol was approved by the ethical review board of the Beijing University of Technology, Beijing, PR.China.

## Discussion

This study has been conducted to cope with the situation caused by the contaminated wastewater drained by firms in industrial areas and to minimise the risk of diseases caused by industrially contaminated wastewater. The WWTPs can be a helpful solution to minimise the health risk of people living in industrial areas. For this purpose, we have presented an integrated model based on the theory of planned behaviour. We have proposed three research questions to answer the research problem. In response to RQ1, we have presented six hypotheses (H1-H6), and for RQ2, we have assessed the H1a-H6a. For RQ3, we performed sensitivity analysis and ranked the influential factors with respect to their normalised importance. We have used PLS-SEM to conclude the proposed hypothesis and understand the moderation effect of firm size on the intentions to install WWTPs.

Environmental, social, and governance criteria, often known as ESG criteria, are a set of standards for a firm's conduct that socially concerned investors use to analyse possible investments. Environmental criteria evaluate a firm based on the measures it takes to protect the environment, taking into account, for instance, the policies it has in place to deal with climate change. The company’s management of its connections with its workers, vendors, consumers, and the regions in which it works is an evaluation of the social factors. Criteria related to the environment could include company climate policy, energy consumption, pollution, waste, animal welfare, and natural resource conservation. The criteria may also be used to analyse any potential environmental hazards a business may be exposed to and how the firm manages such risks. Emissions of greenhouse gases, both direct and indirect, compliance with environmental rules, and the management of hazardous waste are all possible factors to take into consideration.

Environmental knowledge (H1) significantly influences the firm's management intentions to install WWTPs. It indicates that as much as a firm and its management are knowledgeable about the danger of contaminated wastewater and the health risks associated with water pollution as much as they are inclined towards the treatment of wastewater that can be reused in industry and be helpful to minimise the risk of disease caused by contaminated wastewater. Firm size moderates the relationship between EK (H1a) and Intentions to install a WWTP. Big firms have a stronger inclination toward the installation of installing WWTPs. A possible reason can be that they are more aware of the danger and concerned about the reputation of the firm and the responsibility of the firm towards society. They might have more knowledgeable management aware of the situation and consequences of wastewater drain. Our findings align with those of earlier researchers in the domestic sector^3,29,62^.

WWTP installation cost (H2) also positively influences the intentions of installing WWTPs in the firms. The possible reason behind this can be that firms are aware of their responsibility and have knowledge that contaminated wastewater of organisations has a hazardous effect on the soil, society, and human health. If they will not look at their responsibility and will not treat the contaminated water, their legal and moral position in society and the market can be at risk. So firms are not concerned about the cost and have the intention to install water treatment plants. The moderation effect of firm size (H2a) is also significant but negative. It means that small firms care about the cost of installing WWTPs, but large firms are more concerned about their social responsibility, reputation, and law binding. The possible reason behind the negative moderation effect can be the low income of small firms and less education about wastewater hazards in small firms. They might also think that the wastewater of these small firms is not hazardous and are unaware of the danger associated with the contaminated wastewater. The findings are consistent with the earlier researcher who claim that eco-friendly products play a role in the sustainable development of developing countries^25^ or adopting renewable technology^62^. Still, it contradicts the findings of earlier studies that installation cost is a barrier^32^ to adopting new technology or with those who claim that economic factors are insignificant in adopting new technology^26^.

Awareness of risk associated with contaminated industrial wastewater (H3) significantly negatively influences the firm’s management. It means that it restricts management from installing the WWTP directly. It is consistent in terms of moderating effect with previous studies but contradicts in terms of direct influence^35^. The moderation effect of firm size (H3a) plays its role, and large firms are more concerned about wastewater treatment than small firms. Small firms may have a lack of knowledge about the severity of the issue and its impacts. At the same time, large firms perceive the problem counter-wise and are willing to treat their contaminated wastewater to remain in the good books of stakeholders, consumers, and the Government. The logical reason can be that large firms have their customers and shareholders internationally and must follow the rules and regulations set by the Government or local authorities. These stakeholders are more educated and aware of the danger of contaminated wastewater and more concerned about the ecological system; hence large firms pay more attention to their social responsibilities to satisfy their customers, shareholders, and government authorities.

Government incentives (H4) have no significant influence on a firm’s management to treat their wastewater regardless of the firm’s size. The possible reason that apparently can be a potential reason in the case of developing countries, specifically Pakistan, is that governments do not have attractive incentives to motivate firms to treat their waste. It can be because of a lack of funds, seriousness toward water management, or government priorities. It has almost the same results where researchers found that government priorities are more inclined towards providing electricity and other facilities compared to taking care of health^18^. But it contradicts earlier researchers’ findings that government incentives play a significant role in electronic vehicles and innovations^36,37,39^ or intentions to use clean water^29^.

Firm reputation (H5) directly influences firms’ management for installing WWTPs, but firm size does not moderate the relationship (H5a). This implies that firm reputation is an important factor for all firms, regardless of size. It is a well-established fact that all businesses want to improve their reputation for society, customers, other stakeholders, and their growth in a market; that’s why all firms, regardless of their size, consider it an influential factor that motivates them to treat their wastewater. The findings are consistent with the earlier research regarding the customers^40^ and environmental performance^41^, i.e. environmental and social criteria.

Corporate social responsibility (H6) also has a positive influence and is stronger for large firms (H6a). It explains that although corporate social responsibility is an influential factor for both kinds of firms, large firms are more concerned about their social responsibility compared to small firms. It is because large firms have several domestic and international checks on their credibility and international customers, and shareholders are more concerned about the firm’s behaviour towards ecological issues. It also demonstrates the firm’s seriousness towards its production process and resource cost reduction. Corporate social responsibility’s influence on the firm’s willingness to treat its hazardous waste is rarely discussed in previous studies. Study results are somehow consistent with previous studies conducted in other contexts and, to some extent, support their claim that a company's reputation is an intermediary in the relationship between corporate social responsibility and firm performance or risk^42^ and that positive associations exist between corporate social responsibility and firm reputation^43^.

In response to the RQ3, the ANN sensitivity analysis revealed that corporate social responsibility is the most important factor for firms installing contaminated WWTPs. Although the remaining factors were found significant in SEM except for Government incentives, sensitivity analysis revealed that other factors except CSR are relatively less important for firms.

### Theoretical contribution

The study findings have a significant contribution to the existing literature. First, our study is one of the first that investigate the firms' management intention to treat their contaminated wastewater. Second, we have studied the environmental knowledge role as an important antecedent to install WWTPs that have gained less attention in previous studies. Third, our study measures the role of awareness about the risks associated with contaminated wastewater and their influence on the firms' decision-making. Fourth, Firms' reputations and corporate social responsibility are mainly used in economics and financial studies to measure and attract potential investors and enhance the goodwill and firm's image. All these factors have rarely been studied in the context of industrial waste treatment and their influence on coping with diseases and ecological issues caused by manufacturing firms. The fifth researcher has rarely investigated the wastewater treatment trends and motivators in developing countries, but our study exclusively focuses on one of the developing countries to study the issue and provide policy implications for developing countries' governments that can be helpful in attaining United Nations' sustainable development goals for 2030. Six, we have investigated the firm size as moderating factor. This concept is used in measuring Capital Structure-Its Determinants Relations^19^ or dynamic capability perspective^20^. Last but not least, we have implied a dual-stage deep learning methodology to capture the linear and non-linear interaction between variables and rank the variables according to their importance. This methodological contribution is rarely presented in previous studies in the context of wastewater treatment. The variance explained in second stage with the help of ANN (79.5%) is considerably higher then the variance explained by SEM (73.3%) methodology. The RMSE values at second stage are also minimul (0.004 for traing, 0.007 for testing) implies the robustness compatability of ANN model in our study. Hence we also recommend the future researcher to utilise the dual stage deep learning model to conclude the results in similar studies.

### Policy recommendations

Based on the study’s findings, we have some policy recommendations for developing countries’ governments, firm management, and other stakeholders. First and foremost, corporate social responsibility is one of the most important factors; hence we recommend that governments retain a check on all the firms and regularly inspect their wastewater management protocols. Ensure wastewater management at the firm’s level. This has two immediate benefits, 1. Water supply management and water distribution checks can be improved, and water reservoirs can use for alternative supply; eventually, the water scarcity issue can be managed 2. Disease caused by contaminated industrial wastewater can be minimised, and the health standards of the masses can be improved, which will reduce the national health budget.

Subsidies and interest-free loans to firms to install WWTPs can be introduced to facilities and encourage firms to play their role as responsible pillars of the economy. GI is not significant in our study; that is clear evidence that either firms’ do not know about the incentives announced by the Government or it's very few. Hence, we also recommend that governments announce clear incentives and policies on wastewater treatment by firms.

We also suggest conducting industrial wastewater contamination and associated health risk and water waste awareness workshops at the firm level to manage the issue of water scarcity and disease caused by contaminated industrial wastewater.

### Limitations and future research avenues

Our study is limited in some areas; it can be used in future research directions to improve our findings and scope. First, we collected our sample from a single country; wastewater treatment intentions can vary in other geographical boundaries, and governments' incentives role can be different in developing or developed countries. Second, we have collected datasets from manufacturing firms, other sectors' preferences may vary, and future studies can involve the different types of business to validate the findings or cross-compare the study results using cross-comparison between different kinds of business. Thirdly we did not study the firm’s net worth. Future studies can incorporate firms' net worth as an influential factor.

### Supplementary Information

Supplementary Information.

## Data Availability

The dataset used in this study is available on reasonable demand from the corresponding author.
