# Supplementary material for: The role of corporate social responsibility and government incentives in installing industrial wastewater treatment plants: SEM-ANN deep learning approach
Source: Sci Rep. 2023 Oct 2;13:16529. doi: 10.1038/s41598-023-37239-1 (PMC10545828; doi:10.1038/s41598-023-37239-1)
Supplement: Supplementary file 1 — Supplementary Information. [file 41598_2023_37239_MOESM1_ESM.docx]

**Table S1. Cross loadings**

|  | **ARACW** | **CSR** | **EK** | **FREP** | **GI** | **IC** | **IWTP** |
| --- | --- | --- | --- | --- | --- | --- | --- |
| ARACW1 | **0.855** | 0.333 | 0.868 | 0.363 | 0.45 | 0.349 | 0.394 |
| ARACW2 | **0.877** | 0.364 | 0.887 | 0.499 | 0.544 | 0.397 | 0.409 |
| ARACW3 | **0.864** | 0.313 | 0.87 | 0.409 | 0.456 | 0.34 | 0.443 |
| ARACW4 | **0.835** | 0.287 | 0.744 | 0.343 | 0.474 | 0.331 | 0.294 |
| CSR1 | 0.299 | **0.859** | 0.304 | 0.486 | 0.434 | 0.416 | 0.692 |
| CSR2 | 0.36 | **0.893** | 0.362 | 0.589 | 0.466 | 0.387 | 0.71 |
| CSR3 | 0.352 | **0.911** | 0.356 | 0.602 | 0.442 | 0.377 | 0.762 |
| EK1 | 0.855 | 0.333 | **0.868** | 0.363 | 0.45 | 0.349 | 0.394 |
| EK2 | 0.877 | 0.364 | **0.887** | 0.499 | 0.544 | 0.397 | 0.409 |
| EK3 | 0.864 | 0.313 | **0.87** | 0.409 | 0.456 | 0.34 | 0.443 |
| FREP1 | 0.259 | 0.479 | 0.271 | **0.7** | 0.395 | 0.336 | 0.487 |
| FREP2 | 0.391 | 0.512 | 0.389 | **0.826** | 0.434 | 0.376 | 0.489 |
| FREP3 | 0.477 | 0.522 | 0.488 | **0.865** | 0.492 | 0.415 | 0.562 |
| GI1 | 0.497 | 0.492 | 0.493 | 0.522 | **0.872** | 0.375 | 0.455 |
| GI2 | 0.454 | 0.395 | 0.452 | 0.442 | **0.847** | 0.268 | 0.342 |
| GI3 | 0.48 | 0.394 | 0.466 | 0.447 | **0.846** | 0.289 | 0.416 |
| IC1 | 0.33 | 0.394 | 0.336 | 0.431 | 0.295 | **0.886** | 0.499 |
| IC2 | 0.367 | 0.356 | 0.356 | 0.402 | 0.325 | **0.865** | 0.41 |
| IC3 | 0.386 | 0.4 | 0.387 | 0.397 | 0.344 | **0.86** | 0.481 |
| IWTP1 | 0.322 | 0.663 | 0.33 | 0.556 | 0.355 | 0.338 | **0.819** |
| IWTP2 | 0.516 | 0.671 | 0.538 | 0.518 | 0.457 | 0.344 | **0.848** |
| IWTP3 | 0.295 | 0.698 | 0.31 | 0.536 | 0.379 | 0.649 | **0.83** |

**Table S2. Questionnaier**

| **Variables** |  | **Measurement Items** | **References** |
| --- | --- | --- | --- |
| Intention to install a water treatment plant | IWTP1 | Our firm intends to install a water treatment plant. | [1, 2] |
|  | IWTP2 | Our firm is planning to use water treatment plants continuously. |  |
|  | IWTP3 | Our firm will use a water treatment plant in the future. |  |
| Environmental Knowledge | EK1 | I am very knowledgeable about wastewater treatment plants and their related environmental benefits. | [1, 2] |
|  | EK2 | I know that wastewater treatment plants are essential in fighting against water pollution. |  |
|  | EK3 | I know that I buy environmentally safe products as I am more cautious about my health. |  |
| Installation Cost | IC1 | The installation cost of wastewater treatment plants is reasonable. | [2] |
|  | IC2 | The cost our firm will pay for wastewater treatment plants is a good match for its value. |  |
|  | IC3 | At present wastewater treatment plant installation costs deliver a good value. |  |
| Awareness of Risks associated with contaminated water | ARACW1 | I am aware of the risk associated with contaminated water and its use. | [2] |
|  | ARACW2 | I am aware that contaminated water is hazardous to health. |  |
|  | ARACW3 | I am aware that contaminated water causes multiple diseases. |  |
|  | ARACW4 | I am aware that contaminated water needs to be treated before draining. |  |
| Firm Reputation | FREP1 | To what extent do you think installing a wastewater treatment plant will increase your company's goodwill locally and internationally? | [3, 4] |
|  | FREP2 | To what extent do you think the installation of a waste water treatment plant will bring more benefits to your brands? |  |
|  | FREP3 | To what extent do you think installing a wastewater treatment plant will provide an extra edge to your products within the industry over your competitors? |  |
| Government incentives | GI1 | To what extent do you think government provides a subsidy for wastewater treatment plants? | [5] |
|  | GI2 | To what extent do you think government provides tax incentives after the installation of wastewater treatment plants? |  |
|  | GI3 | To what extent do you think government provides enough incentives for installing wastewater treatment plants? |  |
| Corporate social responsibility | CSR1 | To what extent do you think the installation of the wastewater treatment plant is your corporate social responsibility? | [6] |
|  | CSR2 | To what extent do you think installing wastewater treatment plants is necessary to avoid diseases that originate from the contaminated water in society? |  |
|  | CSR3 | To what extent do you think the installation of a wastewater treatment plant can be your contribution to the society where your live in or surrounded by? |  |

**References:**

1. Mustafa, S., et al., *Does Health Consciousness Matter to Adopt New Technology? An Integrated Model of UTAUT2 With SEM-fsQCA Approach.* Frontiers in Psychology, 2022. **13** DOI: 10.3389/fpsyg.2022.836194.

2. Mustafa, S., et al., *Role of eco-friendly products in the revival of developing countries' economies & achieving a sustainable green economy.* Front. Environ. Sci, 2022. **10** DOI: 10.3389/fenvs.2022.955245.

3. Hess, R.L., *The impact of firm reputation and failure severity on customers' responses to service failures.* Journal of Services Marketing, 2008. **22**(5): p. 385-398 DOI: 10.1108/08876040810889157.

4. Khanifah, K., et al., *Environmental performance and firm value: Testing the role of firm reputation in emerging countries.* International Journal of Energy Economics and Policy 2020. **10**(1) DOI: <https://doi.org/10.32479/ijeep.8490>.

5. Gong, S., A. Ardeshiri, and T. Hossein Rashidi, *Impact of government incentives on the market penetration of electric vehicles in Australia.* Transportation Research Part D: Transport and Environment, 2020. **83**: p. 102353 DOI: <https://doi.org/10.1016/j.trd.2020.102353>.

6. Nguyen, N.T.T., N.P. Nguyen, and T. Thanh Hoai, *Ethical leadership, corporate social responsibility, firm reputation, and firm performance: A serial mediation model.* Heliyon, 2021. **7**(4): p. e06809 DOI: <https://doi.org/10.1016/j.heliyon.2021.e06809>.
